# Cristae remodeling causes acidification detected by integrated graphene sensor during mitochondrial outer membrane permeabilization

**DOI:** 10.1038/srep35907

**Published:** 2016-10-27

**Authors:** Ted D. Pham, Phi Q. Pham, Jinfeng Li, Anthony G. Letai, Douglas C. Wallace, Peter J. Burke

**Affiliations:** 1 Department of Biomedical Engineering, University of California, Irvine, CA, USA; 2 Department of Chemical Engineering and Materials Science, University of California, Irvine, CA, USA; 3 Dana-Farber Cancer Institute, Harvard University, Boston, MA, USA; 4 Center for Mitochondrial and Epigenomic Medicine, Children’s Hospital of Philadelphia and Department of Pathology and Laboratory Medicine, University of Pennsylvania, Philadelphia, PA, USA; 5 Department of Electrical Engineering and Computer Science, University of California, Irvine, CA, USA

## Abstract

The intrinsic apoptotic pathway and the resultant mitochondrial outer membrane permeabilization (MOMP) via BAK and BAX oligomerization, cytochrome c (cytc) release, and caspase activation are well studied, but their effect on cytosolic pH is poorly understood. Using isolated mitochondria, we show that MOMP results in acidification of the surrounding medium. BAK conformational changes associated with MOMP activate the OMA1 protease to cleave OPA1 resulting in remodeling of the cristae and release of the highly concentrated protons within the cristae invaginations. This was revealed by utilizing a nanomaterial graphene as an optically clear and ultrasensitive pH sensor that can measure ionic changes induced by tethered mitochondria. With this platform, we have found that activation of mitochondrial apoptosis is accompanied by a gradual drop in extra-mitochondrial pH and a decline in membrane potential, both of which can be rescued by adding exogenous cytc. These findings have importance for potential pharmacological manipulation of apoptosis, in the treatment of cancer.

The intrinsic mitochondrial pathway of apoptosis is an important target for pharmacological manipulation for a variety of diseases including cancer^1^,^2^,^3^,^4^,^5^,^6^,^7^. This pathway is regulated by the BCL-2 family proteins^8^ and results in the collapse of the inner membrane electrochemical gradient. An early step in the initiation of the intrinsic apoptosis pathway is the mitochondrial outer membrane permeabilization (MOMP). MOMP can be induced by BH3-only proteins such as tBid and BIM and has been proposed to result from the oligomerization of pro-apoptotic BCL-2 family proteins BAX and BAK^8^. BAX and BAK oligomerization activates the metalloprotease, OMA1, to cleave the inner membrane protein OPA1^9^. OPA1 tethers the inner membrane cristae loops together at cristae junctions creating the inter-cristae luminal spaces into which the electron transport chain pumps protons during oxidative phosphorylation (OXPHOS)^10^. Cleavage of OPA1 results in remodeling of the cristae and the opening of the proton-rich cristae luminal spaces^9^,^11^. MOMP permits the release of stored inter-membrane space pro-apoptotic proteins including cytochrome c (cytc), procaspase-9, and Smac/DIABLO into the cytoplasm, causing activation of caspases and the commitment to cell death.

It has been reported that the cytosol becomes acidified soon after the intrinsic apoptosis pathway is activated^12^,^13^,^14^. However, there has not been a method to quantify and thus understand the molecular and physiological basis of this phenomenon.

Here, we present an electronic method to detect extra-mitochondrial pH of isolated mitochondria, based on tethering the mitochondria to one-atom thin graphene. The mitochondria are tethered via graphene bound antibodies which recognize the mitochondrial outer membrane protein, TOM20. Graphene is an excellent conductor and changes in the pH surrounding the mitochondria can change the graphene conductance and be detected electrically. Being optically transparent, the graphene layer also permits optical interrogation of the mitochondria^15^,^16^,^17^ concurrent with analysis of ionic changes. Hence, our system permits the simultaneous monitoring of changes in extra-mitochondrial pH through graphene conductance and inner membrane potential (ΔΨ_m_) using the potentiometric fluorescent dye tetramethylrhodamine ethyl ester perchlorate (TMRE).

## Results

**Figure 1:**
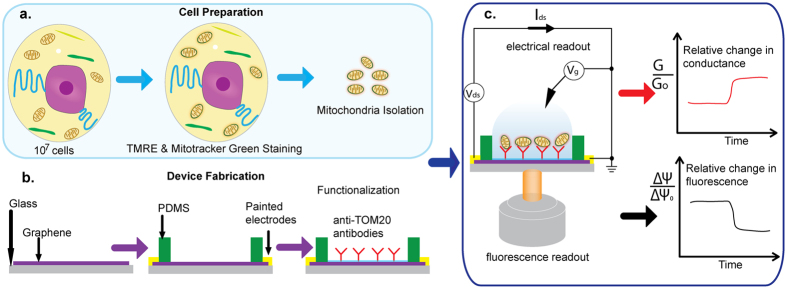
Overview of the experimental workflow. (**a**) Mitochondria are isolated on the day of experimentation and loaded on (**b**) a pre-functionalized device. (**c**) After a brief incubation, mitochondrial functions can be probed via electrical and fluorescent methods.

An overview of our experimental system is shown in Fig. 1. After the graphene device is fabricated and prepared, purified mitochondria can be tethered to the anti-TOM20 antibodies. The graphene conductance then permits the electronic detection of mitochondrial ion exchange and the optical properties of the graphene permit the staining and visualization of the mitochondrial membrane potential.

**Figure 2:**
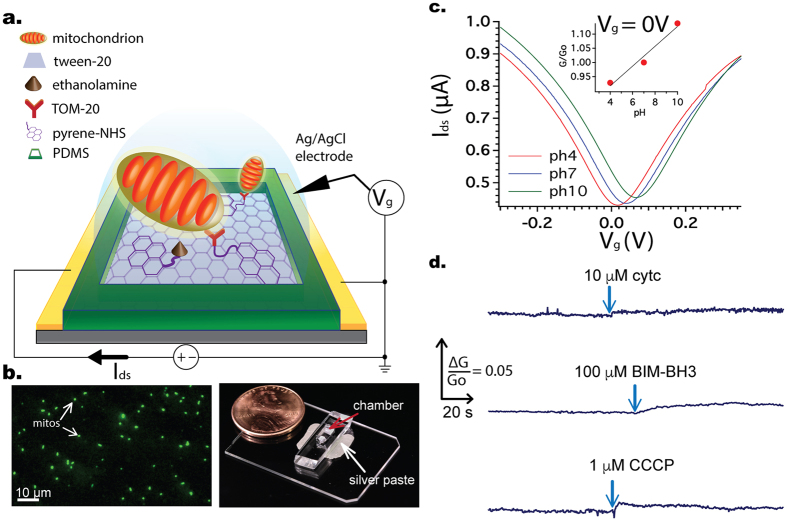
(**a**) Overview of the functionalization scheme (Mitochondria not to scale); (**b**) Immobilized mitochondria (from HeLa cells) on functionalized graphene surface, false colored from 100 nM MitoTracker^®^ Green FM signals. A finished graphene-on-glass device; graphene is at the bottom of the chamber indicated by the red arrow; (**c**) I_ds_ vs V_g_ (electrolyte gate) characteristics with different pH KCl buffers; (**b**). Insignificant fluctuations of graphene conductance following the additions of various substrates.

We used a bottom-up approach to deposit several layers of chemistry on the graphene surface (Fig. 2a). Starting with the base layer of chemical vapor deposition (CVD)-grown single-layer graphene^18^ directly transferred on a glass slide, we incorporated 1-pyrenebutanoic acid succinimidyl ester (pyrene-NHS) as a linker between graphene and anti-TOM20 antibody. While pyrene exhibits a strong pi-pi interaction with the graphene, NHS provides a terminal for amide bonding of antibodies. Since TOM20 is a subunit of the translocase of outer membrane, anti-TOM20 antibody can be used to attract mitochondria^19^. Anti-TOM20 antibodies were incubated and allowed to bond with the pyrene-NHS linker. After antibody incubation, ethanolamine was added to inactivate the NHS remaining ester bonds. Finally, TWEEN20 was added to passivate the exposed graphene area, effectively shielding the exposed graphene surface from unspecific protein adsorption.

After functionalization, the device was washed with KCl buffer (140 mM KCl, 2 mM MgCl_2_, 10 mM NaCl, 0.5 mM EGTA, 0.5 mM KH_2_PO_4_, 2 mM HEPES, 5 mM succinate, 2 μM rotenone, pH 7.2 adjusted with KOH) twice before 40 μl of 0.14 μg/μl isolated HeLa mitochondria (0.07 μg/μl for RS4;11) was incubated in the device’s chamber for 15 minutes at 4 °C. After incubation, the device was gently washed with fresh KCl buffer and then mounted on the inverted microscope. Because graphene was directly transferred on the glass slide, the focusing distance from the objective to the graphene was minimal, allowing visualization of single isolated mitochondria with fluorescence stains such as MitoTracker^®^ Green FM. Figure 2b shows mitochondria, previously stained with MitoTracker^®^ Green FM, are immobilized on top of the graphene layer at the bottom of the device. Gentle insertions of experimental reagents did not disturb the mitochondria as shown in Supplementary Fig. 1. In a similar fashion, a graphene device without anti-TOM20 antibody or just bare graphene was incubated with isolated mitochondria but did not result in any mitochondria attachment on the graphene surface after the wash step.

The density of mitochondria attachment was calculated to be 4237 ± 279 mitochondria/mm^2^ for HeLa cells and 1418/mm^2^ ± 328 for RS4:11 cells; the sample sizes were three. We used ImageJ particle analysis to count the number of mitochondria within 0.25–1 μm^2^ under the assumption that a mitochondrion’s area would fall in this range. Since the chamber’s bottom area is 20 mm^2^, we can compute that a device would have approximately 85,000 mitochondria at the upper limit. If we assumed that 1 μg of mitochondrial protein yields 10^6^ particles of isolated mitochondria^20^, after washing, our device would require at most 0.1 μg of mitochondrial protein. This quantity is much smaller than the number of mitochondria that would be included in more conventional solution biochemistry assays, ranging from 3–20 μg mitochondrial protein for assays of cytc release^21^, single mitochondria membrane potential^22^, and respiration studies^23^. In addition, this work pushes the minimal mitochondrial quantity requirement even further compared with our previous reports^24^,^25^, which used 0.75 μg of mitochondrial protein per assay. Using our typical yield of 140 μg mitochondrial protein from 10^7^ HeLa cells, 0.1 μg of mitochondrial protein amounts to around 7000 cells worth of mitochondria, a slight improvement to the 10,000-cell requirement imposed for plate-based assays of mitochondrial functions^17^.

### Functionalized Graphene devices are pH-sensitive, but insensitive to CCCP, BIM-BH3, and cytc, and are only mildly sensitive to succinate and K^+^ ions

We measured the depletion curves, defined as the graphene current vs. electrolyte gate voltage curve (I_ds_ vs. V_g_), of the functionalized graphene devices with KCl buffer at different pH values. 1 M HCl and 1 M KOH were used to adjust the pH of the KCl buffer from pH 4 to 10. Figure 2c shows that the depletion curve shifts right with increasing pH, and at zero gate voltage the current increases. The gate in these experiments is the electrolyte (buffer) in the chamber. The pH sensitivity of graphene is consistent with previous reports^26^,^27^. From the inset of Fig. 2c, at zero gate voltage an increase in one unit change in pH corresponds to approximately 4% increase in conductance. This calibration, however, only applies to functionalized devices without mitochondria. For the devices with tethered isolated mitochondria, the situation is more complicated and is discussed in Supplementary Fig. 3. Additionally, we carried out a series of control experiments to confirm that the dominant response of the graphene was to pH, and not other chemicals used in the buffer or likely to have been modified by the mitochondria (Fig. 2d. and Supplementary Fig. 11). Having established a relatively low sensitivity of graphene to experimental substrates (as compared to the pH sensitivity), we set out to test the graphene response in the presence of isolated mitochondria.

### CCCP causes ΔΨm to decrease and buffer pH to increase

The mitochondrial proton motive force is defined as Δp = ΔΨ_m_ − 59ΔpH, where ΔΨ_m_ denotes the mitochondrial inner membrane potential and ΔpH indicates the proton gradient across the inner membrane. The mitochondrial inner membrane potential can generally be monitored by lipophilic cations or indicator dyes. We chose 40 nM TMRE^28^ as the membrane potential indicator. The concentration of the charged dyes (in this case, TMRE) changes with membrane potential in accordance with the Nernst equation (1), which states the concentration ratio is given by:


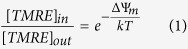


This means at higher potentials (defined as the matrix is more negative), more TMRE accumulates inside the mitochondrial matrix. As a result, TMRE fluorescence intensity from inside the mitochondria can indicate qualitatively the mitochondrial membrane potential i.e. the higher the intensity, the higher the membrane potential. Assessing ΔΨ_m_ quantitatively requires the TMRE intensity outside of the mitochondria^16^,^29^, however, most studies on mitochondrial membrane potential report only the TMRE intensity inside the mitochondria^30^,^31^. In addition to TMRE fluorescence, we performed concurrent electrical detection on the isolated mitochondria using graphene and correlated the electrical signal to the observed fluorescent readout. The experiments were performed in the dark with the excitation LEDs intermittently turned on to monitor TMRE fluorescence. Hence, graphene photo-conductance was minimal and no interference from the LEDs was observed from the recorded electrical signal.

**Figure 3:**
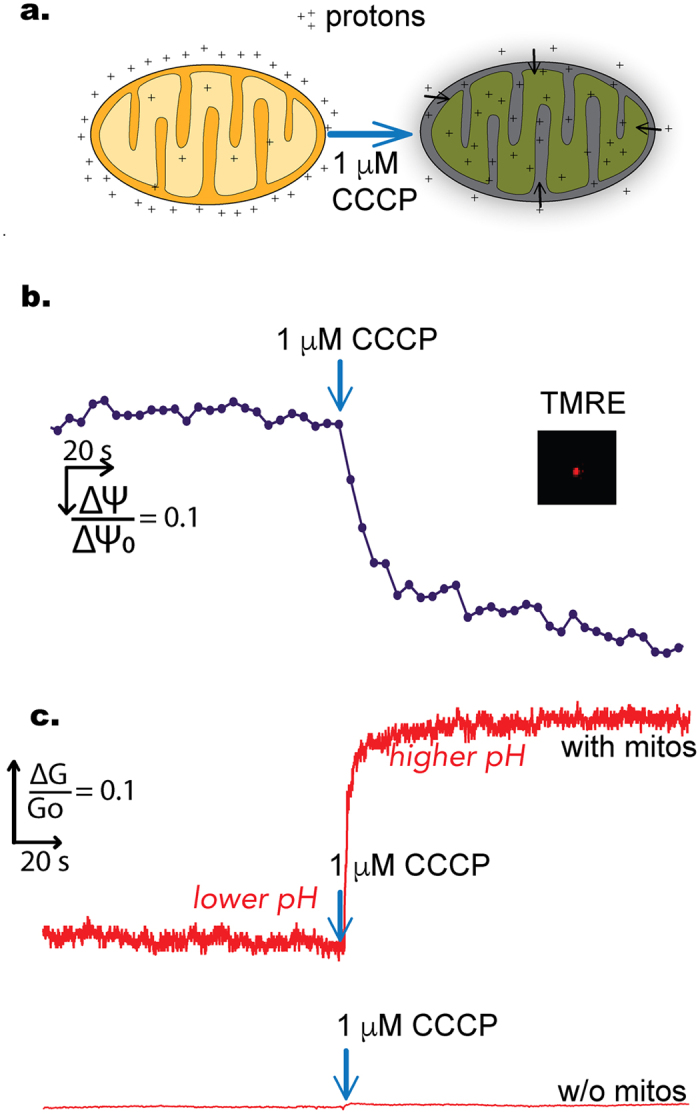
(**a**) Illustrations of protons movement after CCCP addition; (**b**) Time-lapse of TMRE fluorescence, an indicator of the mitochondrial membrane potential, before and after the addition of CCCP; (**c**) The corresponding change in graphene conductance (measured simultaneously with the fluorescence) and the control; after CCCP, the concentration of protons changes dramatically in the extra-mitochondrial buffer.

Figure 3 shows the normalized change in both the graphene conductance and TMRE signal after the addition of 1 μM CCCP, a strong uncoupling agent that can abruptly depolarize the membrane potential. CCCP works as an efficient proton transfer agent that effectively short circuits the proton (pH) gradient as well as the membrane potential (charge gradient). Compared to the starting fluorescence, after addition of CCCP, the fluorescent signal, defined as TMRE intensity of one mitochondrion, dropped about fifty percent. At the same time, the graphene conductance increased by almost twenty percent, indicating a buffer pH increase.

The results for the CCCP experiment were reproduced five times yielding a statistics of increased conductance at 13 ± 8%. The average reduction in TMRE intensity after CCCP addition was 69 ± 7% (N = 3). In addition, we also performed the same experiment but with another, albeit weaker, uncoupling agent namely 10 μM 2,4-Dinitrophenol and observed a similar change in electrical and fluorescence signals (Supplementary Fig. 4). Meanwhile, the control electrical experiments, performed on the same day with the other experiments on identically fabricated devices but without any mitochondria, yielded at most 0.8% difference in graphene conductance.

In order to confirm the alkalization of the buffer on CCCP addition, and to rule out the possibility that the graphene pH response was an artifact of the relatively novel and new graphene pH detector in the context of tethered mitochondria, we used a traditional, commercial pH meter to measure the pH change in a mitochondrial suspension. The response time of the pH meter was around 3 seconds. We diluted 200 μg isolated HeLa mitochondria in 2 mL of KCl buffer and measured the pH with an Oakton Instruments pH meter and an Orion^TM^ ROSS pH electrode. Before CCCP, our buffer pH was 7.21 ± 0.01 which increased to 7.23 ± 0.01 after CCCP (N = 3). Although this measurement was right at the limit of the sensitivity of the commercial pH meter, the results were consistent with our much more sensitive graphene results.

### BIM-BH3-induced MOMP causes oligomycin-independent buffer acidification (↓ pH_buffer_) and ΔΨ_m_ decay

We utilized BIM-BH3 peptide (derived from BIM protein, see Methods for sequence) as a known activator of BAX/BAK to induce MOMP. In whole cells, BAX is soluble in the cytosol while BAK is present on the outer membrane. In our experiments, because we used isolated mitochondria where only BAK is present and no BAX in the buffer, BIM-BH3 could only affect BAK. Previous studies^5^,^8^,^32^ have demonstrated that BIM-BH3 can directly induce BAK homo-oligomerization, which forms pores in the mitochondrial outer membrane causing MOMP, leading to the release of cytc and other pro-death factors with a side effect being the eventual collapse of the inner membrane potential. The experiments by Letai and colleagues^5^ tested BIM-BH3 on wildtype and BAK deficient liver mitochondria and demonstrated that BIM-BH3 activates BAK oligomerization. Because BIM-BH3 can cause MOMP, one potential concern is that BIM-BH3 can disrupt the binding between the antiTOM20 antibodies and the mitochondria, thus releasing the mitochondria away from the graphene surface. This possibility is unlikely, however, since BIM-BH3 can integrate into the outer membrane without TOM20^33^.

**Figure 4:**
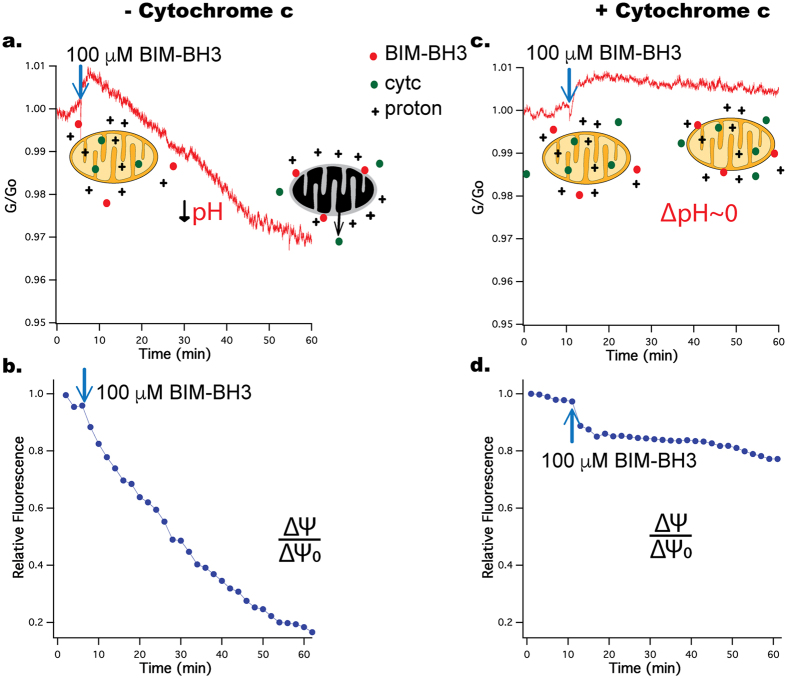
(**a,b**) Addition of 100 μM BIM-BH3 causes graphene conductance and membrane potential to decrease. The fluorescence signal was defined as the average of three mitochondria. Results are representative of three independent experiments. (**c,d**) A similar experiment with 100 μM BIM-BH3 but with 10 μM cytc added to the buffer. The results show less reduction in both graphene conductance and membrane potential. Results are representative of two independent experiments.

Also for the experiments with BIM-BH3, we chose RS4;11 isolated mitochondria due to their well-demonstrated outer membrane composition of BCL-2 family proteins particularly BAK^34^. Addition of CCCP to RS4;11 mitochondria caused a comparable increase in buffer pH as seen for HeLa cell (Supplemental Fig. 5). Upon introduction of 100 μM BIM-BH3 to the RS4;11 mitochondria, we observed a distinct acidification of the buffer (measured via decreased graphene conductance, Fig. 4a), a result confirmed with HeLa mitochondria (Supplementary Fig. 6), as well as a decline in the membrane potential (Fig. 4b). Both of these effects were observed with and without 2 μg/ml oligomycin (which blocks the transport of protons by ATP synthase) in KCl buffer (Supplementary Fig. 8). The induced pH changes following BIM-BH3 addition occurred over a longer timescale than that resulting from CCCP exposure.

Again, as a check to confirm that the measured acidification was not an artifact of the novel graphene biosensor, we measured the buffer pH with a commercial pH meter on introduction of BIM-BH3 to a mitochondrial suspension (140 μg of RS4;11 mitochondria in 500 μL total volume) under otherwise identical conditions. Our measurements showed an average pH reduction of 0.05 ± 0.01 (N = 3) after 60 min of incubation with the peptide.

### Exogenous cytc blocks buffer acidification and rescues ΔΨm after BIM-BH3 induced MOMP

During MOMP, cytc is released to the buffer so the lack of cytc results in loss of cytc mediated electron transfer between complex III and IV in the electron transport chain (ETC). This stalls the ETC, leading to a gradual decline in membrane potential and loss of respiration^35^,^36^,^37^. Consistent with this hypothesis, following MOMP, respiration can be restored by adding exogenous cytc^36^,^37^,^38^. Motivated by this observation, we decided to test the effect of exogenous cytc on buffer acidification and membrane potential in our system. The presence of cytc in the buffer does not affect the graphene sensor (Fig. 2d).

**Figure 5:**
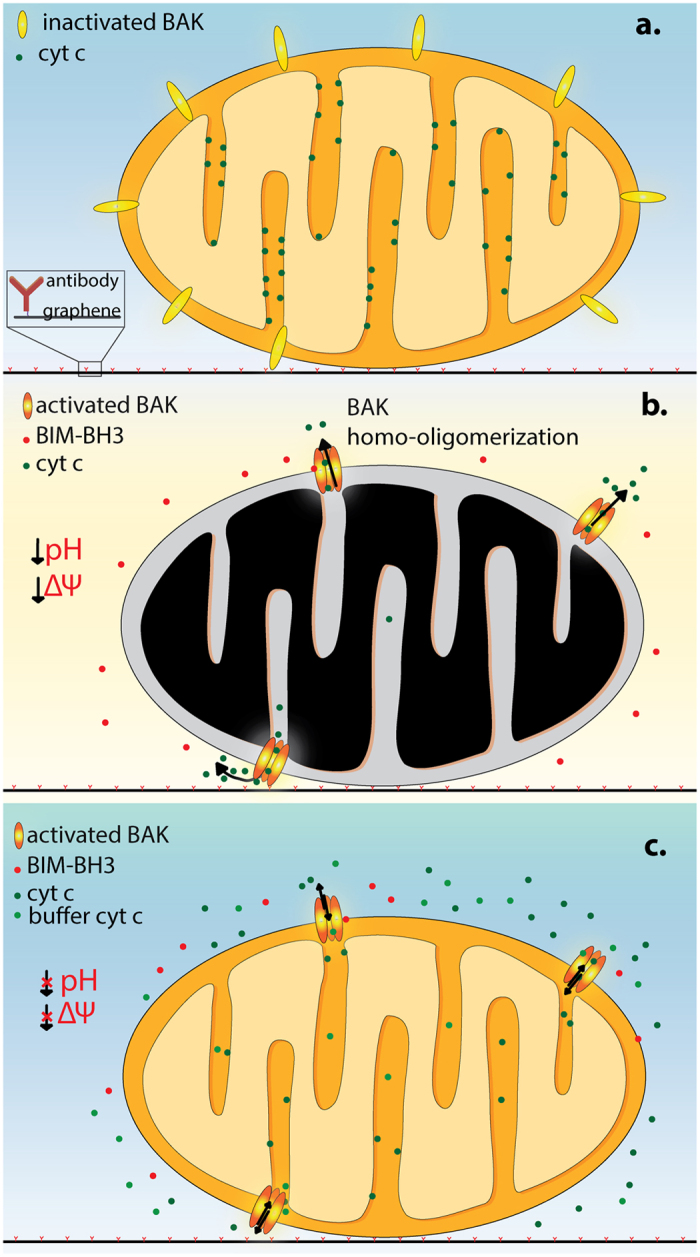
BIM-BH3-induced MOMP in tethered, vital, isolated mitochondria. The mitochondrion (0.5–1 μm in diameter) is drawn to scale with the antibody (~10 nm). (**a**) without BIM-BH3, BAK stays inactive; (**b**) with BIM-BH3, BAK is activated and oligomerize to form pores in the outer membrane, causing cytc release. This is observed to result in buffer acidification and membrane potential decline; (**c**) With exogenous cytc in the buffer, the mitochondrion maintains inner membrane potential with a reduced change in the buffer pH.

We tested the effect of including 10 μM cytc in the buffer on the graphene signal upon treating the mitochondria with 100 μM BIM-BH3 (Fig. 4c,d). The addition of exogenous cytc following BIM-BH3-induced MOMP blocks the decline of TMRE fluorescence (or ΔΨ_m_*) and also greatly reduces the effect of buffer acidification*. To our knowledge, *this inhibition of buffer acidification by exogenous cytc following MOMP is a new observation for isolated mitochondria*. Separate pH measurements of mitochondrial suspensions in the presence of cytc confirm this observation, showing only 0.02 ± 0.01 reduction in buffer pH with cytc as compared to 0.05 ± 0.01 (p < 0.05 student t-test, N = 3) in the case without cytc. Figure 5 summarizes our experimental observations without any hypothesis about the mechanisms.

## Discussion

We have created a novel system for assessing functional changes in mammalian mitochondria. Our system employs an optically transparent, single atom thin graphene layer onto which the mitochondria are tethered using a graphene-bound antibody against the outer membrane protein TOM20 to which isolated mitochondria adhere. Because the graphene electrode is optically transparent, single bound mitochondria can be functionally analyzed using analytical fluorescent dyes at the same time that the mitochondrial outer membrane can be monitored in real time by changes in the conductance of the graphene. The electrical properties of the graphene have been found to detect subtle changes in the proton concentration on the outside of the mitochondrial outer membrane as the mitochondrial inner membrane electrochemical gradient changes. The acute sensitivity of this system is permitting the dissection of alterations in the mitochondrial membrane status during various mitochondrial processes such as uncoupled depolarization of OXPHOS and the induction of the intrinsic pathway of apoptosis initiated by the pro-apoptotic peptide BIM-BH3.

Using our graphene biosensor platform, we have been able to make three important observations pertaining to the mitochondrial inner membrane electrochemical gradient and its changes during the BIM-BH3 initiated intrinsic pathway of apoptosis. First, we found that CCCP addition to respiring mitochondria causes buffer alkalization along with membrane potential depolarization. Second, we observed that treatment of respiring mitochondria with BIM-BH3 results in external buffer acidification and membrane potential decline. Third, we found that the BIM-BH3-induced acidification and membrane potential decline of respiring mitochondria can be blocked by the addition of exogenous cytc to the external mitochondrial buffer.

**Figure 6:**
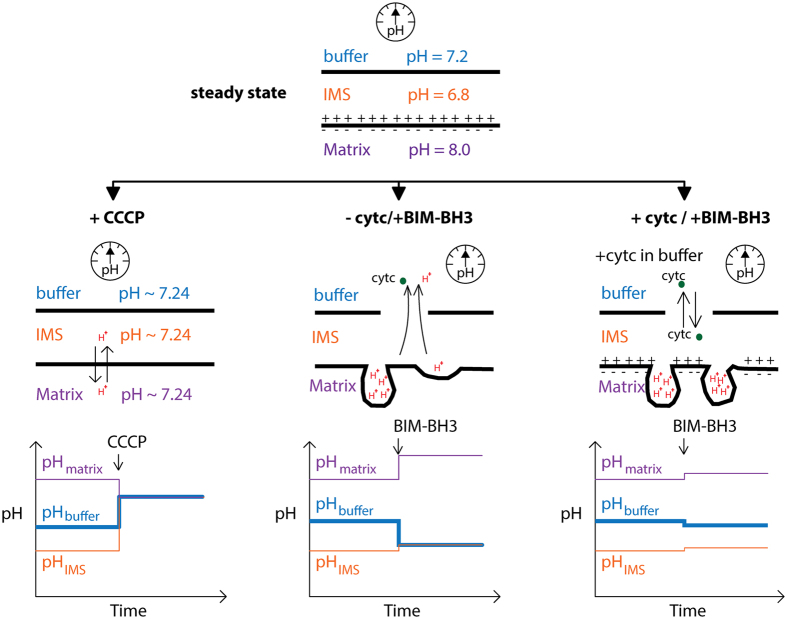
Schematic diagrams of membrane morphology and compartmental pHs for the three experiments performed. The steady state of the mitochondria is shown at the top with buffer pH (indicated as measured) and the other pHs as inferred from the literature^61^,^62^. In the case of CCCP, pH is equilibrated across the three compartments. The bottom shows the qualitative changes in pH vs. time (not to absolute scale) with the bolded line being the measured variable while the other lines are inferred. For the next two cases, the top two figures correspond to our proposal that the cristae remodel and release protons and cytc from the invaginations and the absence of such a mechanism when exogenous cytc is added. The bottom figures indicate again the qualitative assessment of the pHs.

Our first observation of buffer alkalization can be attributed to CCCP allowing protons to rapidly enter the matrix. When immersed in isotonic buffer containing the mitochondrial respiratory chain complex II substrate, succinate, and the complex I inhibitor, rotenone, mitochondria rapidly oxidize the succinate passing the electrons from complex II, to coenzyme Q, to complex III, to cytc, to complex IV, and finally to O_2_ to reduce it to H_2_O. The flux of electrons through complex III and IV is coupled to the transport of protons from the mitochondrial matrix out across the mitochondrial inner membrane to the inter membrane space rendering the outside of the mitochondrial inner membrane acidic and positively charged and the inside negatively charged and alkaline^39^. Addition of uncouplers to the respiring mitochondria result in the rapid flux of protons back into the mitochondrial matrix, rendering the inter-membrane space more alkaline^40^,^41^,^42^ (Fig. 6). Given that the mitochondrial outer membrane voltage dependent ion channel^43^ (VDAC or porin) is freely permeable to protons this would result in the pH of the surrounding buffer to become more alkaline, exactly as we observed, a result consistent with Mitchell’s chemiosmosis theory^44^ for oxidative phosphorylation (OXPHOS) coupling.

We propose that buffer acidification with BIM-BH3 in our second observation is caused by release of protons from the lumina created by the invaginations of the mitochondrial inner membrane. At first glance, the acidification of the surrounding mitochondrial buffer on treatment of respiring mitochondria with the inducer of the intrinsic pathway of apoptosis, BIM-BH3, would seem contrary to the chemiosmosis theory of OXPHOS. However, recent discoveries^9^,^11^,^45^ about structure and function of the mitochondrial inner membrane and the changes that occur during the induction of the intrinsic pathway of apoptosis render the observation of acidification explicable.

The mitochondrial inner membrane is highly invaginated and with the tips of the invaginations closest to the outer membrane held together by the mitochondrial inner membrane integral membrane protein OPA1^10^ located within the mitochondrial contact site and cristae organizing system (MICOS). This creates multiple internal lumina in which the interior of each lumen is equivalent to the outside of the mitochondrial inner membrane. Hence, within these lumina the cytc and proton concentration can become very high.

When the mitochondrial membrane potential declines or the intrinsic pathway of apoptosis is activated mitochondrial inner membrane proteases including OMA1 become activated and cleave OPA1 resulting in the opening of the inner membrane lumina to the mitochondrial intermembrane space^9^,^11^. Since most of the cytc is encompassed within the lumina of the inner membrane^46^,^47^ this process is central to the release of cytc from the mitochondria observed as the intrinsic pathway of apoptosis progresses. Because protons are much smaller in diameter than cytc, it follows that as OPA1 is cleaved, the first event is the escape of the protons into the mitochondrial inter membrane space and out through the permeabilized outer membrane and into the surrounding buffer (Fig. 6). This would cause the gradual acidification of the surrounding medium detected by the graphene electrodes. Treatments of the mitochondria with BH3 peptides often require 60–90 min incubation^17^,^48^ before any functional measurements can detect a change. This time frame is consistent with the completion of the pH change that we detected with the graphene sensor which occurred at about 60 minutes.

Given that the decline in the mitochondrial inner membrane potential is an important prerequisite for the activation of inner membrane proteases and the cleavage of OPA1, it follows that the release of cytc from the mitochondrial inter membrane space following BIM-BH3 induced BAK activation could locally activate the proteases that cleave OPA1. This possibility follows from the conceptualization of the distribution of the respirasomes across the mitochondrial inner membrane. The respirasomes would fall into two categories: those that are located on the mitochondrial inner membranes that form the cristae invagination-generated lumina and pump protons into the cristae lumina and those respirasomes that are located in the inner membrane which faces the intermembrane space. This later set of respirasomes would be responsible for maintaining the pH acidification of the intermembrane space and thus would control the activation of the outer membrane proteases that can cleave OPAI. When BIM-BH3 activates BAK to open the outer membrane, the first cytc to be released would be that in the inter membrane space resulting in localized drop in the mitochondrial membrane potential, activating OMA1, and initiating the cleavage of OPA1. Once OPA1 is cleaved the invaginated cristae lumina would open to the inner membrane space and the remaining cytc would be released stopping all further respiration. Addition of exogenous cytc to the medium surrounding the mitochondria would sustain the respiration required for maintaining the intermembrane space pH and membrane potential thus stabilizing OPA1 and blocking detectable changes in proton and cytc release (Fig. 6), which is our third observation.

Although previous studies have shown that cytosolic acidification is associated with apoptosis in whole cells^12^,^13^,^49^, its role, timing, and mechanism has remained until now a mystery. One proposed mechanism (ATP synthase reversal^14^) has been ruled out on thermodynamic grounds^50^. Our observation of acidification even in the presence oligomycin (which blocks ATP synthase activity) also rules out ATP synthase involvement. Furthermore, our results provide a plausible mechanism that is consistent with recent discoveries of OMA1/OPA1 based cristae remodeling, Although it is generally believed that mitochondria remodel during apoptosis^51^, to directly measure cristae structure during apoptosis is technically challenging, and beyond the scope of this work. Our hypothesis that cristae remodeling causes buffer acidification is the most consistent hypothesis with our data, as well as all of the data in the literature on this subject^51^.

Furthermore, it is still highly plausible that the physiological role of pH change is to enhance caspase activities during apoptosis, as the caspases are known to have pH-dependent activation^49^. These questions have led to a “renaissance” of interest in mitochondrial pH^52^. In addition, because the tumor microenvironment also exhibits significant pH alterations, studying mitochondrial mediated pH changes would make for fruitful future studies.

As with any new bio-assay technology, it is important to benchmark results against known standards, and to compare and contrast advantages and disadvantages of the new vs. traditional methods. For pH sensing, there are two traditional approaches: Electrochemical sensors and genetically encoded pH sensitive fluorescent proteins such as pHluorin, SypHer, and pHred. We have chosen to benchmark our results against a conventional pH meter (Oakton Instruments pH meter and an Orion^TM^ ROSS pH electrode), and found the results to be consistent. We found this to be more expedient than engineering a separate strain with pH sensitive fluorescent proteins. However, the conventional pH meter has barely sufficient pH resolution (0.01 pH) and a slow response time, as compared to our integrated graphene detectors. Consequently, we had to use significantly more sample (140 μg vs 0.1 μg) with the pH meter, and probably because of 0.01 pH resolution we could only detect pH changes after 60 min of mitochondrial incubation with BIM-BH3. In contrast, the intimate contact between the graphene and the mitochondria allows our graphene devices to detect an estimated 4.0 × 10^−4^ relative change in pH unit (Supplementary Information) with 0.2 s temporal resolution. The enhanced sensitivity comes with a drawback that the pH calibration for the graphene devices is not absolute (Supplementary Information).

In principle, a genetically engineered mitochondrial pH sensor based approach has the advantage that an absolute calibration can be performed for pH sensing. However, this approach has resulted in some uncertainties in the literature about the proton motive force dynamics during apoptosis^14^,^49^. Still, recent advances in live cell imaging have enabled detailed (and calibrated) studies of mitochondrial and cytosolic pH under different metabolic conditions^53^,^54^. While advantageous in many ways, genetically encoded mitochondrial pH sensors do have some disadvantages, including restricted applicability to established transformed cell lines.

What our ultrasensitive graphene system for analyzing isolated mitochondria has revealed is a model where there are two totally different mitochondrial inner membrane electrochemical gradients. One of these is the proton gradient between the mitochondria matrix and the intermembrane space. This gradient appears to be an important mediator for the regulation of OMA1 and the resulting regulation of OPA1 cleavage, the important factor in linking the opening of MOMP with the initiation of the intrinsic pathway of apoptosis. However, the second inner membrane electrochemical gradient is between the mitochondrial matrix and the enclosed lumina of the cristae invaginations which are isolated from the intermembrane space electrochemical gradient by closure of the cristae lumina by MICOs and OPA1. This structure potentially permits a much higher concentration of protons to be generated, since the pH cannot be buffered by the cytoplasm surrounding the mitochondrion. Furthermore, by confining the protons in a small volume, the electrostatic potential can be much higher increasing the energetic capacity of the system.

In conclusion, by taking advantage of our ability to study isolated mitochondria using our graphene sensor, we have been able to separate two fundamentally different classes of mitochondrial inner membrane, respirosomes, and electrochemical gradients, resulting in a strikingly new perspective of the role of the mitochondrial electrochemical gradient and thus of the biophysics associated with the Mitchell’s chemiosmosis hypothesis^44^.

The development of the graphene-based system for monitoring changes in the mitochondrial function permitted us to investigate events associated with mitochondrial OXPHOS coupling and the intrinsic pathway of apoptosis. However, we have only begun to monopolize on the exquisite time and sensitivity resolution that our new system permits. Further miniaturization of the technology from millimeter to micrometer dimensions (a straightforward process using photolithography) could enable assays on single mitochondria. In addition to the studies presented herein, this platform could enable new studies of mitochondrial biology and medicine that were heretofore inaccessible. The first possibility is the parallelization from one to thousands of on-chip devices. Such a system could permit for the first time the analysis of 1) mitochondrial heterogeneity within a single cell, say as mtDNA mutations in heteroplasmy^55^ leading to functional heterogeneity, an unexplored frontier in mitochondrial biology; 2) massively parallel screening for peptides and drugs that could modulate mitochondrial respiration or the intrinsic pathway of apoptosis. For example, it has recently been shown that mitochondrial sensitivity to apoptosis/MOMP/cytc release in response to chemotherapies in tumor biopsies (*in vitro*) is directly correlated with patient survival rate^56^. Further investigation has led to a proposed “profile” of each tumor, defined as its qualitative response (defined as cytc release) to a panel of peptides, entitled “BH3 profiling”^17^. Another potential application is high temporal resolution, high pH resolution studies of mitochondrial flashes^54^,^57^. The role of these flashes, and their relationship to signaling pathways, metabolism, bioenergetics, apoptosis and the mitochondrial permeability transition pore is only now beginning to be understood.

## Methods

### Graphene transfer and device fabrication

Graphene was transferred on to glass substrates using a modified protocol from what we described previously^18^. Briefly, a 5 cm × 5 cm copper foil containing CVD grown single-layer graphene on one side was cut into 0.6 cm × 1.0 cm sheets. The side of the sheet containing graphene was pressed lightly against a block of pre-cured PDMS. The Cu-graphene-PDMS structure was then placed and left floating in a Cu-etchant bath (50 mg/ml ammonium persulfate in DI water). After the copper was completely etched away, the graphene-PDMS structure was washed three times with DI H_2_O for one hour to eliminate any residual ions from the copper etching step. The wet PDMS/graphene block was then pressed against a 1-mm thick glass slide that had been cleaned for one hour with 1:3 (v/v) H_2_O_2_:H_2_SO_4_ solution. The glass-graphene-PDMS slide was kept under a slight pressure overnight to promote graphene-glass adhesion and allow the interfacial water to evaporate. When the device was completely dry, the PDMS was carefully peeled off, leaving large-area single-layer graphene on the glass slide. Although many transfer steps were essentially similar to the protocol^18^, the deposition of graphene directly on glass was the modification in the study.

Following graphene transfer, we needed to create an experimental chamber on top of the graphene, fabricate drain and source electrodes to measure the graphene in-plane conductance, and insulate the electrodes from the experimental liquid. To accomplish these goals, a simple, freshly cured PDMS slab with an inner cut-out was placed directly on top of the graphene (Fig. 2b). Specifically, the cutout, measured 4 mm × 5 mm × 3 mm in length × width × height, serves as the experimental chamber. Additionally, due to having narrow width, the PDMS slab, although covering most graphene area, still leaves some exposed graphene where quick-dry silver paste (Ted Pella) can be painted to establish electrical connection. As depicted in Fig. 2b, the silver paste electrodes are conveniently insulated from the experimental chamber.

### Graphene functionalization

After the silver-paste electrodes were completely dry, graphene functionalization was carried out using a series of solution deposition, incubation and wash. Each step in the series employed 50 μL volume. First, 3.81 mg of pyrene-NHS was mixed with 2 mL dimethylformamide (DMF), and added to the PDMS chamber of each graphene device. Incubation with pyrene-NHS ensued for one hour at room temperature. The device was then washed sequentially with fresh DMF, DI water and PBS pH 7.2. Anti-TOM20 antibody (Santa Cruz Biotechnology) solution is added at 33.3 μg/mL concentration and incubated overnight at 4 °C. After two wash steps with PBS then DI H_2_O, 0.1 M ethanolamine diluted in DI H_2_O was added and incubated for one hour at room temperature then washed with DI H_2_O. The next incubation with 0.1% TWEEN-20 was set for one hour at room temperature to deactivate the exposed graphene area by preventing unspecific protein adsorption. The functionalization scheme was partially adapted from^58^. Finally, the devices were washed with DI H_2_O then KCl buffer for immediate use.

While the fabrication and functionalization steps might at first seem straightforward, our device yield was around fifty percent. The limiting factor here was the bonding strength between the final PDMS and glass slide, which often resulted in leakage at the DMF step or the overnight incubation at 4 °C. DMF is an organic solvent, which might affect the integrity of PDMS; hence, we remedied by taking extreme care not to spill the solvent anywhere outside of the chamber. Furthermore, we attributed the failing rate at 4 °C step to the humidity inside the refrigerator, so putting the device in a desiccator did increase our yield. We consider fifty percent yield was reasonably robust for our experiments because we would have at least three working (with electrical connections and no liquid leakage) devices on the days of the experiment.

### Cell culture and mitochondria isolation

#### Cell Preparation

The mammalian cell lines: HeLa and RS4;11 (American Type Culture Collection) were maintained in the log growth phase using the appropriate tissue culture protocols for adherent and suspension cells, respectively. All cell culture related supplies such as media, fetal bovine serum, PBS pH 7.2, mitochondria staining dyes and trypsin were obtained from Life Technologies. On the days of the experiment, 10^7^ cells were typically harvested for mitochondria isolation. We used more cells if we needed more mitochondria.

#### Mitochondria Staining

Before isolation, the confluent cells were stained with 100 nM MitoTracker^®^ Green FM and 40 nM TMRE for 1 hour. Subsequent isolation steps used solutions with 40 nM TMRE because TMRE is a potentiometric dye, which fluctuates in and out of the mitochondria depending on their inner membrane potential. 40 nM was determined as the optimal concentration for our experiments (Supplementary Fig. 7).

#### Mitochondria Isolation

Our complete isolation buffer contains 225 mM mannitol, 75 mM sucrose, 0.5 mM EGTA, 20 mM HEPES, 0.5% (w/v) BSA, 1X protease inhibitor, pH 7.2 with 1 M KOH. All chemicals were purchased from Sigma Aldrich. The stock isolation buffer was prepared without BSA and the protease inhibitor and stored at 4 °C. Mitochondria from the cultured cells were isolated using differential centrifugation. After collection, the cells were transferred to a glass homogenization tube in 3 mL of complete isolation buffer and homogenized with 30 strokes on ice for HeLa cells and 40 strokes for RS4;11. The cells were then transferred into 2-ml Eppendorf tubes and centrifuged at a low speed of 2000x g for 4 min at 4 °C. The resulting supernatant was collected and centrifuged at a high speed of 12,000x g × 10 min at 4 °C. After this step, the supernatant as well as the light-colored fluffy sediment containing damaged mitochondria were aspirated and the resulting pellet was resuspended in KCl buffer (140 mM KCl, 2 mM MgCl_2_, 10 mM NaCl, 0.5 mM EGTA, 0.5 mM KH_2_PO_4_, 2 mM HEPES, 5 mM succinate, 2 μM rotenone, pH 7.2 adjusted with KOH). However, for mitochondria protein analysis, the mitochondria were resuspended in KCl buffer without EGTA. The protein analysis was done with BCA assay kit supplied by Thermo Fisher Scientific. A typical mitochondrial preparation with this protocol exhibits a respiratory control ratio of 3.1 (measured with a Hansatech Oxytherm), which is good for cultured cells^59^.

### Fluorescence measurement

Using an Olympus IX71 inverted microscope with two LED excitation sources (490 nm & 565 nm), we observed the red and green fluorescence signals from MitoTracker^®^ Green FM and TMRE. Our field of view from our 20X objective is 200 μm × 300 μm, and we monitored the fluorescence from this field of view by controlling the microscope with NIH Micromanager software, which also set the exposure time to one second.

To process and analyze the images, ImageJ software was used. For the entire field of view analysis, we measured the fluorescence intensity from the field of view. In contrast, for single mitochondrion analysis, we defined regions of interest enclosing the mitochondria and three more identical copies of that region of interest to define the background noise. We then assigned the fluorescence signal of the mitochondria as the measured signal subtracting the noise average.

### Experimental design and electrical measurement

After mitochondria isolation, we loaded about 6 μg of mitochondrial protein of isolated mitochondria to a graphene device and incubated the device for 15 min at room temperature. Following two gentle washes with KCl buffer, the device was secured on the microscope stage, and the focus was adjusted. We then attached electrical contacts to the drain and source terminals of the devices using nickel (type) probes. The gate electrode was a Ag/AgCl electrode. An Agilent sourcemeter B2902A was used to apply the drain-source and gate voltages and to measure the currents. When both optical focus and electrical connection were satisfactory, we proceeded with taking MitoTracker^®^ Green FM images and I_ds_ versus V_g_ curve, and then started both the electrical measurement of I_ds_ versus time and the fluorescence time-lapse measurement of TMRE signal. The electrical data sampling rate was 200 ms/point. In contrast, fluorescence images were taken once every 5 s for the experiments with CCCP or every 2 min for the experiments with BIM-BH3.

The starting volume of our chamber was 45 μL. During the experiment, we added 4.5 μL of the appropriate experimental substrate to achieve its final concentration. Extreme care was taken not to disturb the gate electrode. CCCP and cytc were obtained from Sigma Aldrich. BIM-BH3 peptide (sequence: MRPEIWIAQELRRIGDEFNA) was purchased from Synpeptide with a confirmation of >95% purity.

Control experiments were carried out in a similar manner with the only difference being the lack of isolated mitochondria. IGOR software was used to analyze all electrical data and the fluorescence data that were exported from ImageJ software.

Our KCl buffer contains 5 mM succinate as a carbon source for the mitochondria, facilitating the maintenance of ΔΨ_m_). Since the electrons derived from succinate doesn’t go through complex I, we also added rotenone. Our choice of succinate and rotenone aimed to limit ROS production by complex I^60^. We also intentionally left out ADP in our respiration buffer to avoid ATP synthase activation. In other words, we maximized ΔΨ_m_, maintaining a functional but not functioning ETC, and also limiting ROS production by complex I.

## Additional Information

**How to cite this article**: Pham, T. D. *et al*. Cristae remodeling causes acidification detected by integrated graphene sensor during mitochondrial outer membrane permeabilization. *Sci. Rep.* **6**, 35907; doi: 10.1038/srep35907 (2016).

**Publisher’s note:** Springer Nature remains neutral with regard to jurisdictional claims in published maps and institutional affiliations.

## Supplementary Material

Supplementary Information
