# Supplementary material for: Cristae remodeling causes acidification detected by integrated graphene sensor during mitochondrial outer membrane permeabilization
Source: Sci Rep. 2016 Oct 27;6:35907. doi: 10.1038/srep35907 (PMC5081517; doi:10.1038/srep35907)
Supplement: Supplementary Information [file srep35907-s1.pdf]

SUPPLEMENTARY INFORMATION

Cristae remodeling causes acidification detected by integrated graphene sensor during mitochondrial outer membrane permeabilization

Ted D. Pham[1], Phi Q. Pham[2], Jinfeng Li[2], Anthony G. Letai[3], Douglas C. Wallace[4], Peter J. Burke[1,2,5]

[1]Department of Biomedical Engineering, University of California, Irvine, CA, USA
[2]Department of Chemical Engineering and Materials Science, University of California, Irvine, CA, USA
[3]Dana-Farber Cancer Institute, Harvard University, Boston, MA, USA
[4]Center for Mitochondrial and Epigenomic Medicine, Children's Hospital of Philadelphia and Department of Pathology and Laboratory Medicine, University of Pennsylvania, Philadelphia, PA, USA
[5]Department of Electrical Engineering and Computer Science, University of California, Irvine, CA, USA
Correspondence should be addressed to P.J.B. (email:pburke@uci.edu)

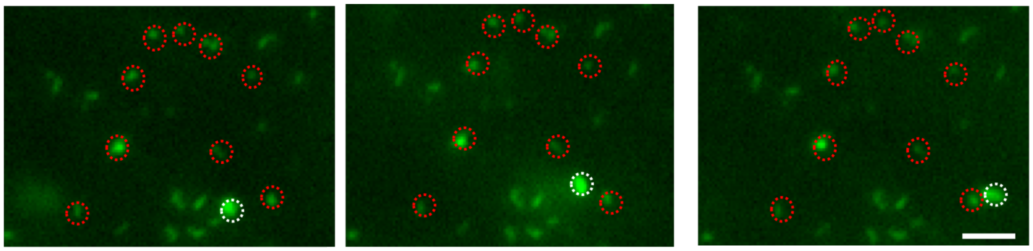

t=0 t= right after 1 uM CCCP t= after CCCP

Supplementary Figure 1: Mitochondria, stained with MitoTracker→ Green FM, on graphene. Red circles indicate some immobilized mitochondria; the white circle denotes one mitochondrion moving during the experiment. Unattached mitochondria are thus considered negligible. MitoTracker→ Green FM is not potential sensitive. Scale bar is 5 μm.

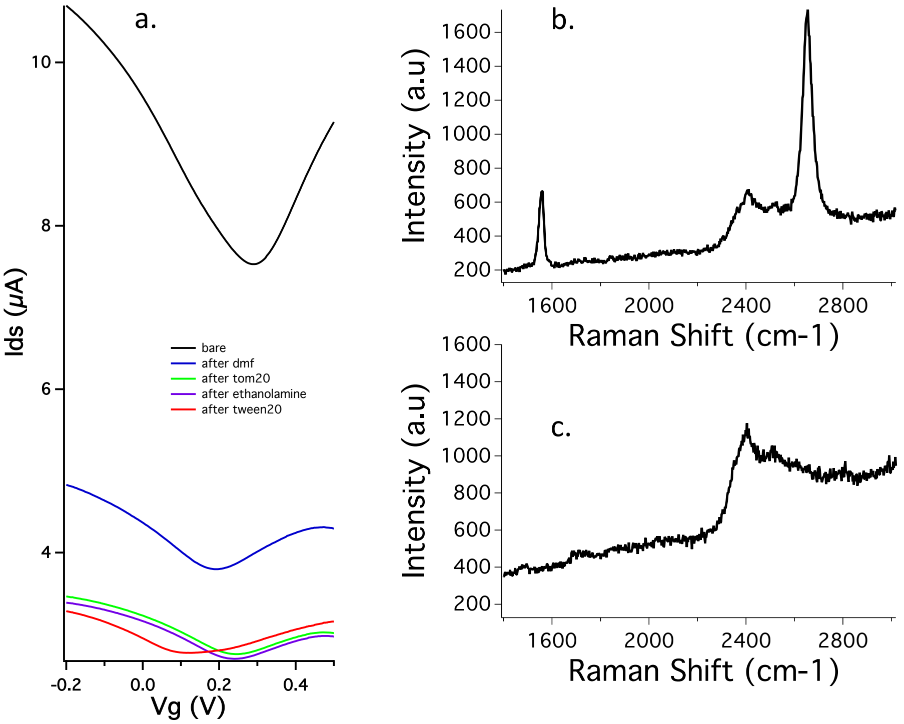

Supplementary Figure 2: (a) Graphene characterization after each functionalization step; (b) Raman spectrum confirms single-layer graphene; (c) Raman spectrum of the glass substrate explains the irregular peak around 2400 1/cm in (b)

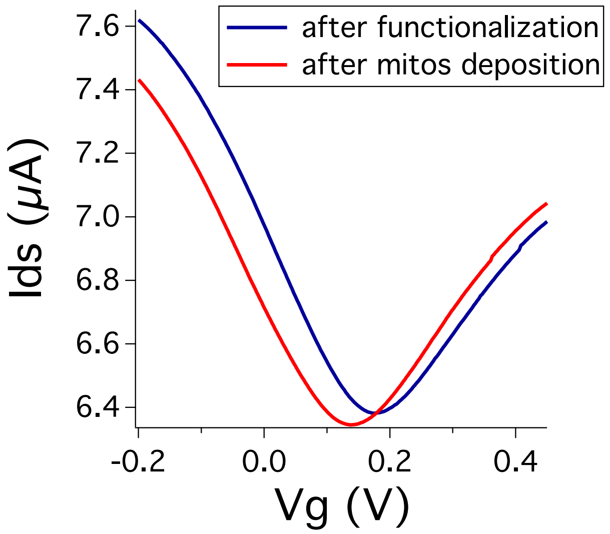

Supplementary Figure 3: Mitochondria deposition shifts the depletion curve. This effect prevents the absolute calibration of pH and renders the calibration on functionalized devices without mitochondria inapplicable to calculating the exact pH change. In addition, exposing the mitochondria to different pH solutions for calibration would adversely affect the mitochondrial functions. Results representative of four experiments.

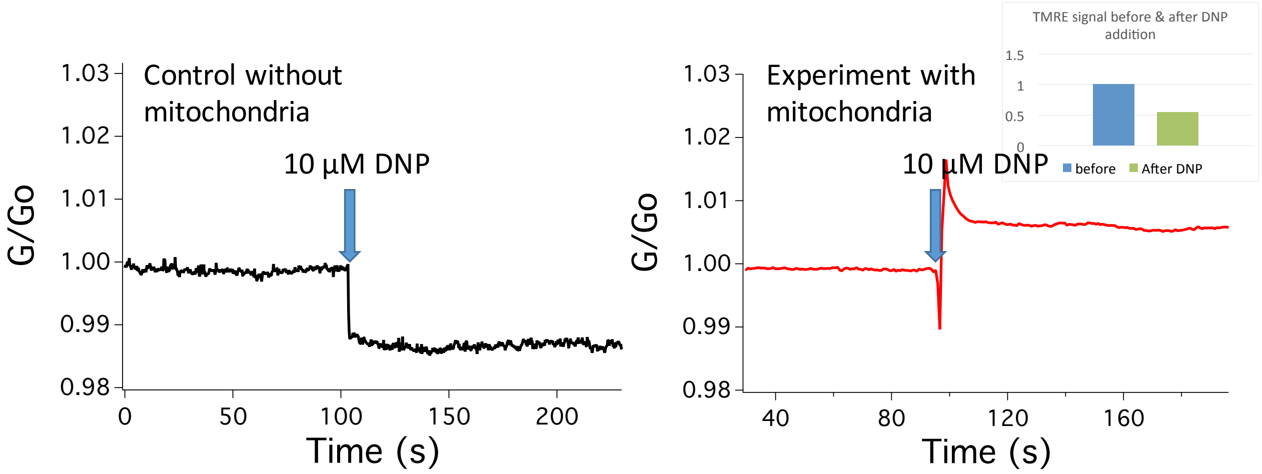

Supplementary Figure 4: Adding 10 µM 2,4-Dinitrophenol (DNP) causes the graphene conductance to increase (Red curve). This effect is expected because DNP is an uncoupling agent similar to CCCP. The inset shows the corresponding loss of membrane potential through TMRE signal. Without mitochondria, however, DNP reduces the conductance by one percent. We think this reduction is due to DNP's low pKa of 4.114. This experiment was done once. HeLa cells were used.

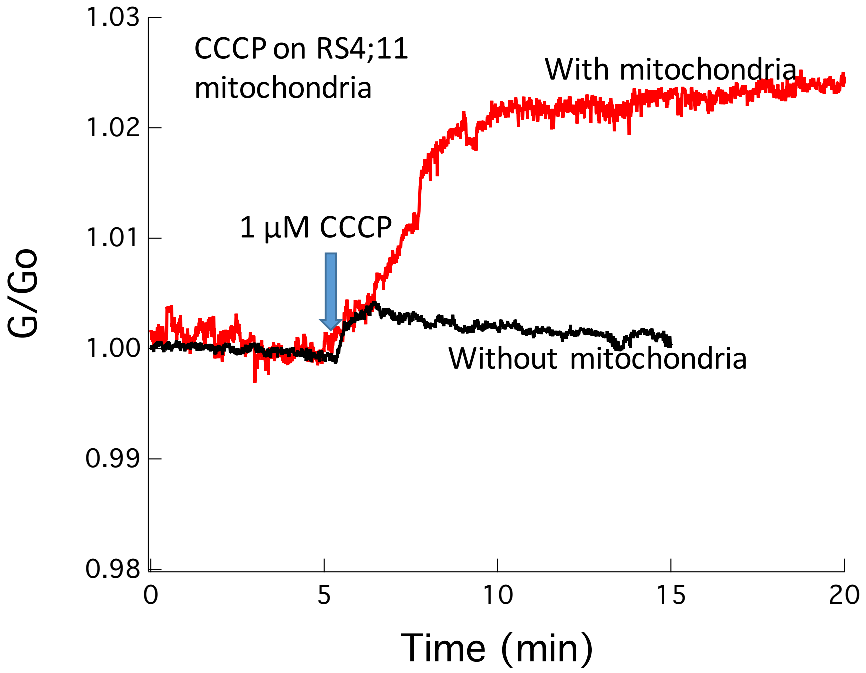

Supplementary Figure 5: Adding 1 µM of CCCP to isolated RS4;11 mitochondria causes the graphene conductance to increase. The increase is relatively smaller than in the case of HeLa mitochondria. This is probably due to the lower quantity of mitochondria obtained from RS4;11. This experiment was done once.

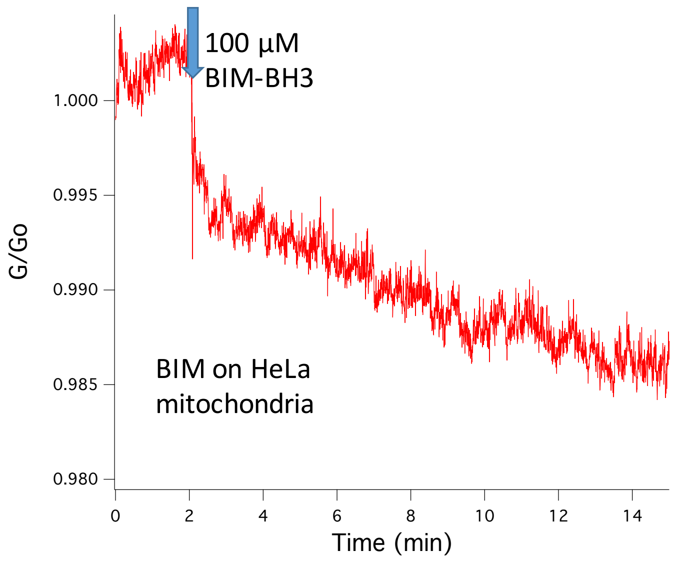

Supplementary Figure 6: Adding BIM to isolated HeLa mitochondria also causes a gradual decrease in the graphene conductance. This experiment was done once. The buffer also contains 2 µg/mL oligomycin.

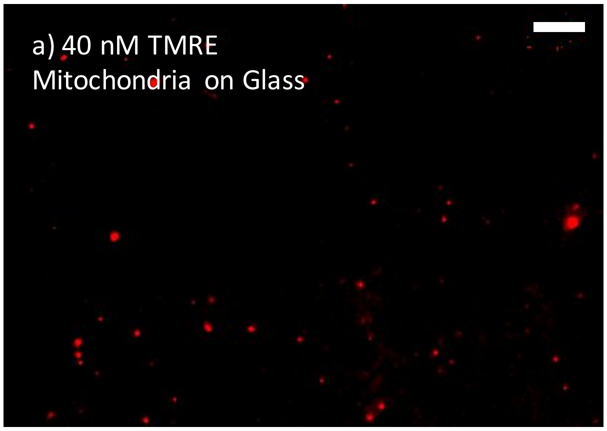

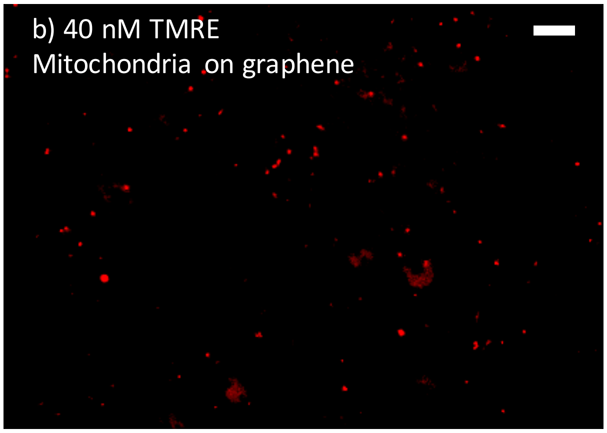

Supplementary Figure 7: We compare 40 nM TMRE stained mitochondria on glass a) and on graphene b); scale bars are 10 µm, false colored red in ImageJ. The contrast level was adjusted to show mitochondria are clearly visible above the background. The signal to noise ratios in both cases are comparable.

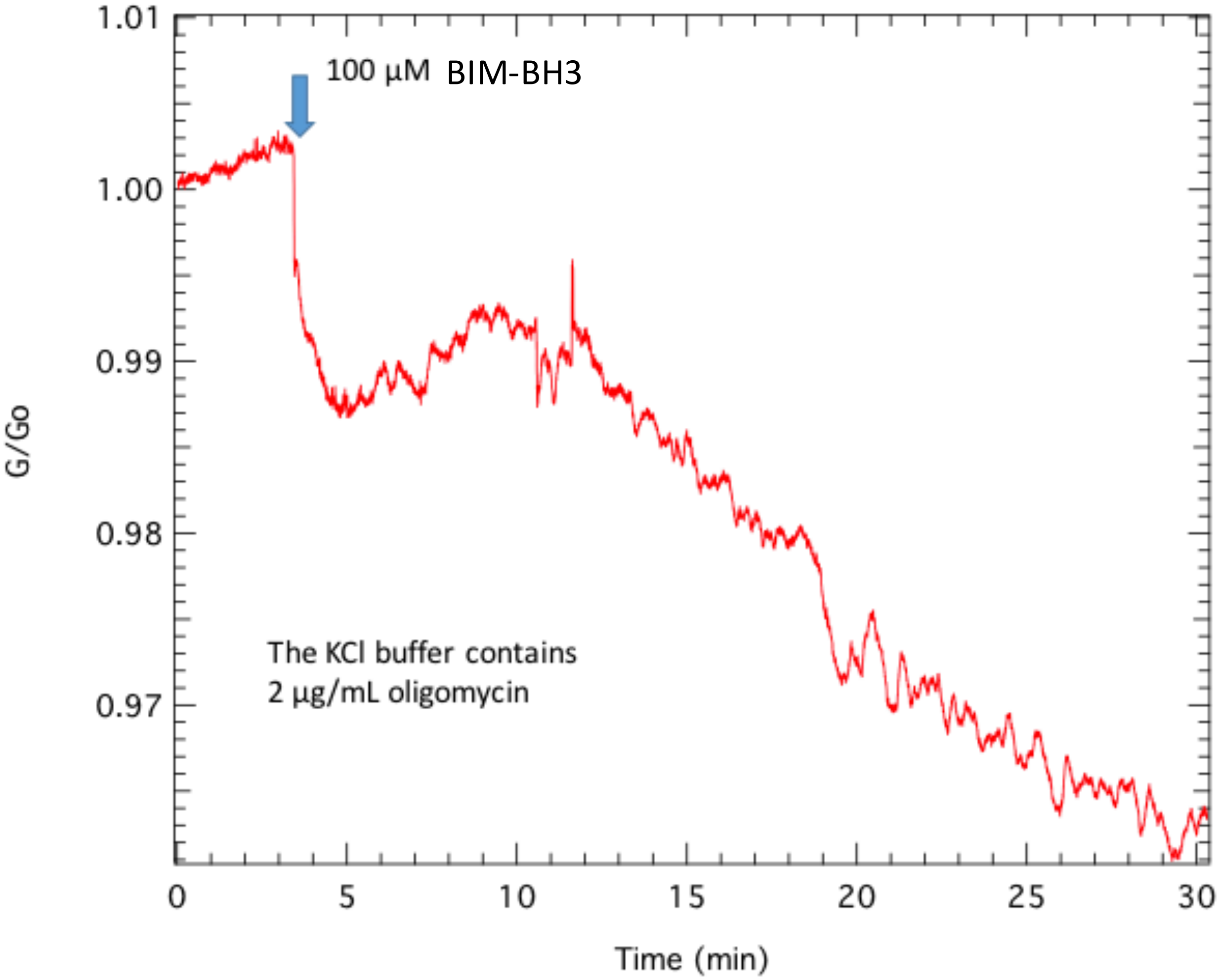

Supplementary Figure 8: Inclusion of 2 µg/mL oligomycin (which blocks ATP synthase) in the KCl buffer does not not affect acidification. Mitochondria were isolated from RS4;11 cells. This experiment was done once. Similar results are shown in Supplementary Figure 6 for HeLa mitochondria.

Supplementary Discussion 1: pH change estimation

A detailed estimate of the pH change measured using the graphene electrode with tethered mitochondria shows that our devices are much more sensitive than commercial pH meters. The intimate contact between the mitochondria and the graphene leads to dramatically enhanced sensitivity of the graphene conductance to small changes in pH, even above and beyond the sensitivity in the “bulk pH change” calibration curve (Fig. 2c). Therefore, the sign of the conductance change indicates acidification or alkalization, but the magnitude of the conductance change (although not quantitatively calibrated) is a much more sensitive measurement of pH than existing commercial pH meters (see below).

Estimate of # of protons per mg of mitochondria taken up during CCCP induced depolarization:

Based on the commercial pH meter measurement, the measured pH change in the bulk suspension of mitochondria can be used to determine the # of protons taken up per mg of mitochondria upon introduction of CCCP.

We apply the Henderson-Hasselbalch equation and account for the movement of protons. In the case of CCCP where a net loss of protons to the mitochondrial inner membrane space, the equation is as follows:

$$pH = pKa + log\frac{[A^-]}{[HA]} \qquad (1)$$

$$\Rightarrow \quad \Delta pH = log\frac{n_{A^-}+n_{H^+}}{n_{HA^-}\, n_{H^+}} - log\frac{n_{A^-}}{n_{HA}} \qquad (2)$$

$$A^-: weak\ acid$$

$$HA: conjugate\ base$$

$$n_{HA}: original\ moles\ of\ the\ weak\ acid\ and\ its\ conjgate\ base$$

$$n_{H^+}: moles\ of\ protons\ loss\ from\ the\ buffer$$

The KCl buffer contains 2 mM HEPES as the majority buffer with a pKa of 7.5. The original buffer pH is 7.2. In the bulk suspension experiments, 200 µg of mitochondrial protein in 2 mL volume induces a pH change from 7.21 to 7.23 after the addition of CCCP (as measured by the commercial pH electrode). Hence, the number of protons transferred to the inner membrane space is 20.7 pmoles/0.1µg.

In the tethered mitochondria experiments, we used 0.1 µg of mitochondrial protein in a 50 µL volume. If we estimate the # of protons taken up based on the bulk measurements above, such

a quantity of mitochondria would induce a pH change of ~$4x10^{-4}$ in the entire 50 µL solution volume, much too small to be measured by commercial pH electrodes. Such a change would also give rise to a small conductance change in the graphene if it occurred in the bulk solution volume (estimated to be 0.001% change from Fig. 2c). However, because of the intimate contact between the graphene and mitochondria, a much larger (few %) change in graphene conductance is measured, indicating the exquisite sensitivity of our devices to mitochondrial induced pH changes compared to existing techniques. A similar analysis in the case of BIM induced release of protons can be carried out, and yields similar conclusions.

Supplementary Discussion 2: pH Resolution

Other pH sensors based on field effective sensing and nano-material have been demonstrated[1–3]. From the Nernst equation, the theoretical sensitivity of a pH sensing surface is around 60 mV/pH. Our computed sensitivity is 8 mV/pH. This limit is, however, not directly related to the detection limit of the platform. The detection limit cannot be computed without accurate noise measurements[4]. To complicate that matter further, while higher sensitivity in terms of mV/pH is generally desired for higher pH resolution, lower mV/pH lowers the charge threshold for detection[5]. Collectively, while the pH sensitivity of semiconducting materials and graphene are promising in biosensing applications, the criterion to assess their performance is still a matter of further research.

Supplementary Discussion 3: The quantity of mitochondria loaded vs. the quantity estimated in each assay

It is worth noting that we used 40 µl of 0.14 µg/µl mitochondrial protein (determined by BCA protein analysis) of HeLa isolated mitochondria. The quantity of mitochondrial protein loaded to a graphene device was then ~5.6 µg. After the incubation and wash steps, unbound mitochondria were certainly washed away so we estimated the mitochondrial protein that was tethered by using the density of particles observed from MitoTracker→ Green FM fluorescence. We claimed this estimated quantity (0.1 µg in the report) is the quantity used by one experiment even though we loaded a higher quantity at the beginning. We would insist that this claim is reasonable because that was the actual quantity of mitochondria used by one experiment. The loading of isolated mitochondria at the beginning can be optimized and we suspect that loading 0.1 µg would yield similar amount of tethered mitochondria requiring a slightly altered parameter such as loading time.

Supplementary Discussion 4: The role of cardiolipin peroxidation during MOMP in buffer acidification

A second order effect concerning cardiolipin peroxidation may contribute to and enhance the observed buffer acidification, although its relative contribution to the effect is hard to estimate quantitatively. Cardiolipin is a proton trap, and hence may serve as a “reservoir” of protons at the inner membrane[6]. However, during MOMP, peroxidation of cardiolipin[7] can release its

trapped protons to the intermembrane space and hence the buffer, causing additional buffer acidification.

Supplementary Discussion 5: Concentrations of peptides

For the majority of the experiments, the peptide concentration was kept at 100 µM. Although this concentration is standard for BH3 profiling method[8,9], due to the amount of assayed mitochondria in this study (100 pg), peptide BIM-BH3 (at 100 µM = 9 µg) may oversaturate the outer membrane and the graphene surface. Therefore, we tested 400 nM of BIM-BH3 (0.036 µg) in inducing MOMP in HeLa mitochondria.

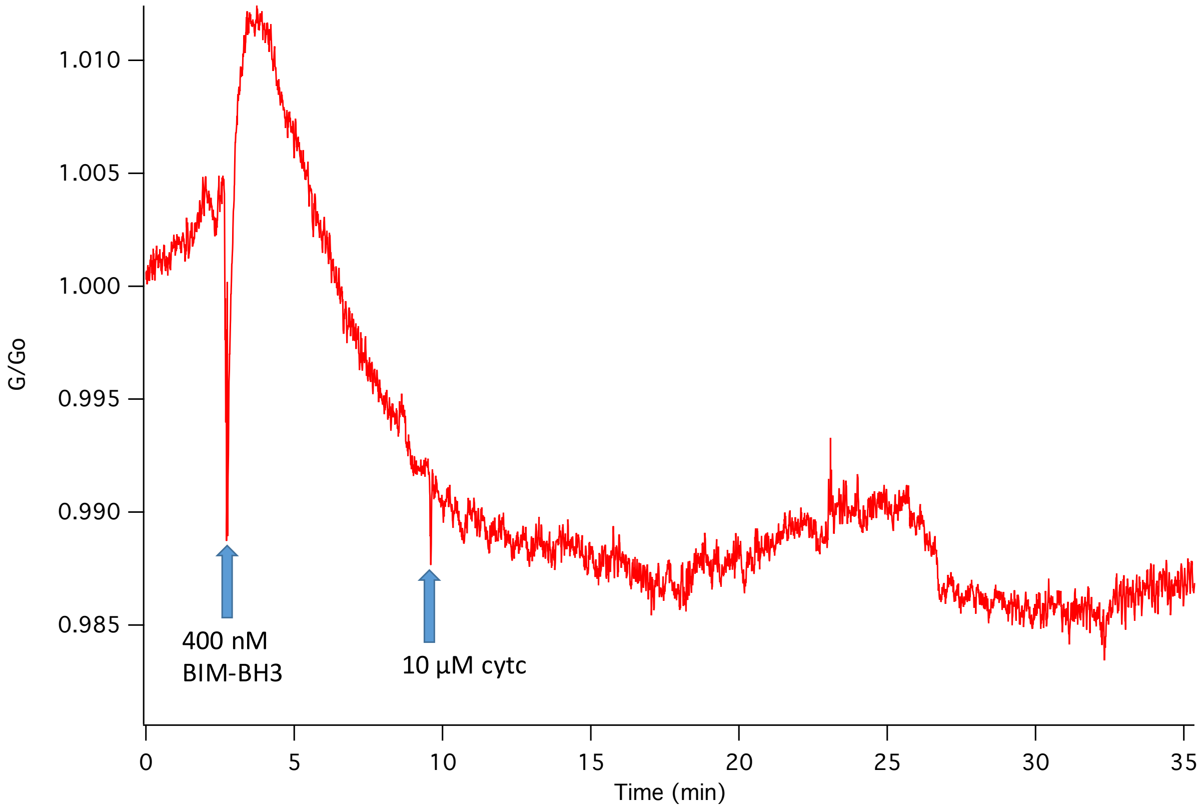

Supplementary Figure 9: Addition of a lower concentration of BIM (400 nM) still reduce the graphene conductance, indicative of acidification. Addition of external cytc stabilized the conductance, evident of loss of acidification. HeLa cells mitochondria were used in this experiment.

Supplementary Figure 9 shows that 400 nM BIM is still effective in reducing the graphene conductance, suggesting acidification.

Supplementary Discussion 6: exogenous cytc added before or after BIM addition has no effect on acidification

Supplementary Figure 9 shows the results where we added exogenous cytc after BIM addition to check if we could rescue acidification. After cytc, the graphene conductance reduced at a slower rate and eventually fluctuate around a straight line, indicative of acidification rescue. Similarly, in Figure 4b of the main text, we showed rescue of graphene conductance decrease (or acidification) when exogenous cytc had been included in the experimental buffer.

Supplementary Discussion 7: ROS production does not affect graphene conductance

Our experimental buffer contained 5 mM succinate as a carbon source for the mitochondria and we also included limit rotenone to block ROS production by complex I. Nevertheless, complex III is also a major source of ROS, which could affect our pH measurements. When cytc is released following MOMP blocked complex III is stalled and thus can start to produce ROS. To investigate if ROS produced by complex III has any effect on acidification, we used 45 nM Antimycin A to our KCl buffer to block complex III. Supplementary Figure 10 shows that inclusion of Antimycin A did not block the conductance reduction by BIM-BH3.

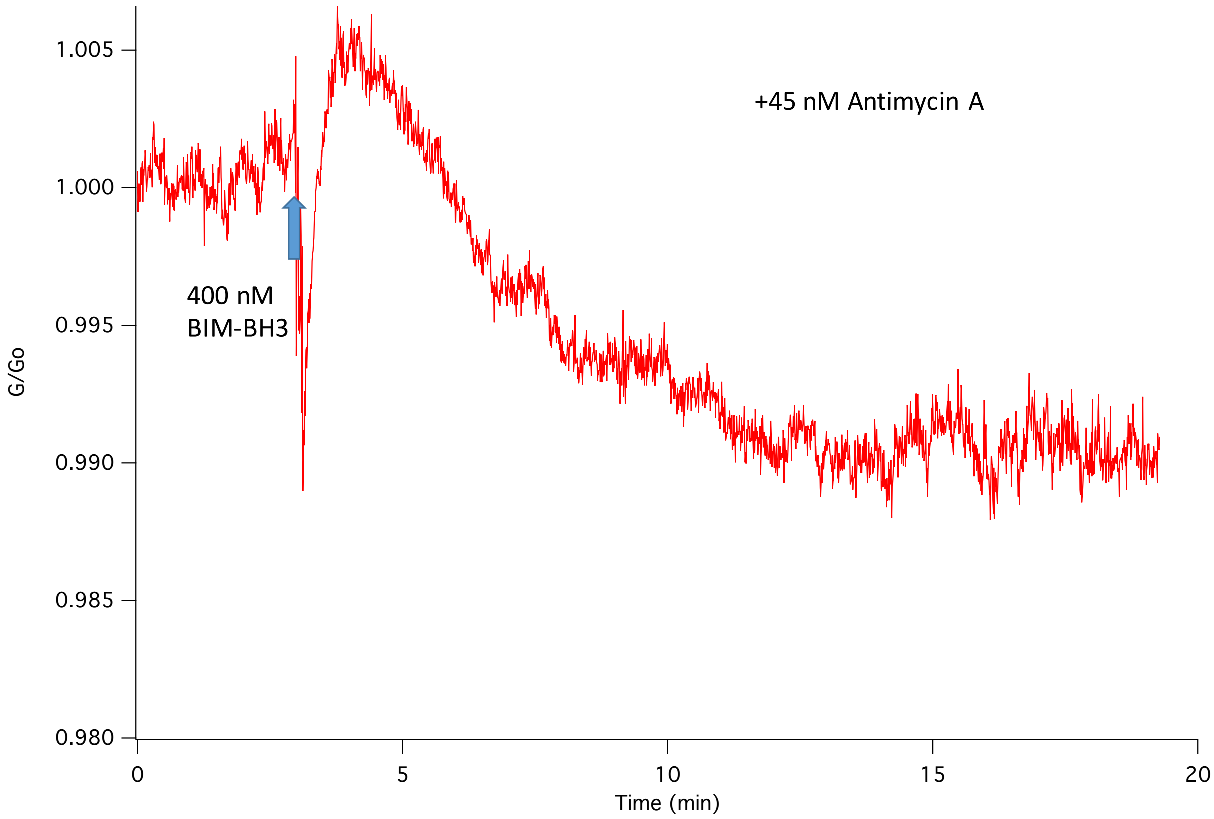

Supplementary Figure 10: Inclusion of 45 nM antimycin A in the experimental buffer did not affect the onset of graphene conductance decline (or acidification). HeLa cells mitochondria were used.

Suppementary Discussion 8: Functionalized graphene sensitivity to other ions other than protons

In Fig. 2d, we showed that the graphene's conductance stable following the addition of 1 μM CCCP, 100 μM BIM-BH3, and 10 μM cytc (final concentrations). Additionally, mitochondria exchange and consume charged species with the buffer, which in principle may also cause changes in the graphene conductance. In particular, potassium fluxes likely occur during mitochondrial permeabilization, while succinate is a weak acid whose diffusion and consumption could contribute to the local pH changes. Therefore, we tested the response of our functionalized graphene to potassium and succinate (Supplementary Fig. 11). For potassium, at 0 V gate voltage, a concentration change of order 100 mM is required to change the graphene conductance by 10%. Because potassium concentration in our experimental buffer is 140 mM, it is unlikely that the potassium fluxes during mitochondrial permeabilization caused any significant current change. Similarly, for succinate, we found that a 10 mM change in succinate concentration would be required to change the graphene conductance by 10% (Supplementary Fig. 11). In our respiration buffer, the mitochondria were kept in State 4 (no ADP, so very little consumption of substrates is expected). Therefore, the consumption of succinate is unlikely to have caused significant change in graphene conductance or local pH. Although it is impossible with existing technology to estimate (or even identify) all of the charged metabolic substrates released by permeabilized mitochondria, because of the low sensitivity of graphene to potassium and succinate, we can assume that other metabolites also do not change the graphene conductance significantly.

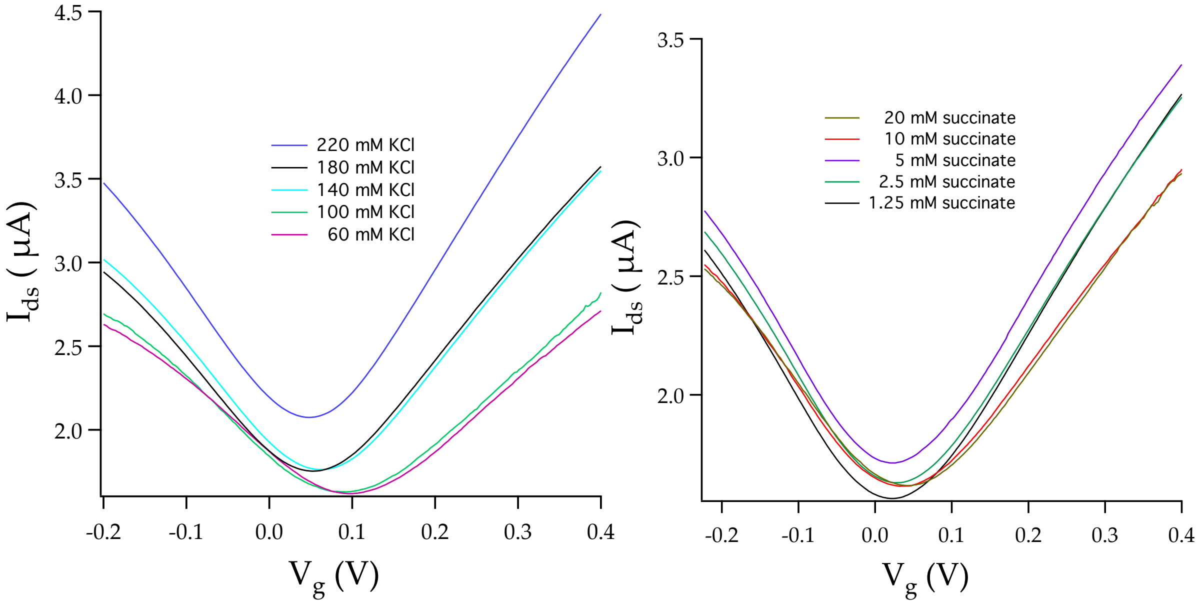

Supplementary Figure 11: For potassium, at 0 V gate voltage, a concentration change of order 100 mM is required to change the graphene conductance by 10%. Similarly, for succinate, we found that a 10 mM change in succinate concentration would be required to change the graphene conductance by 10%.

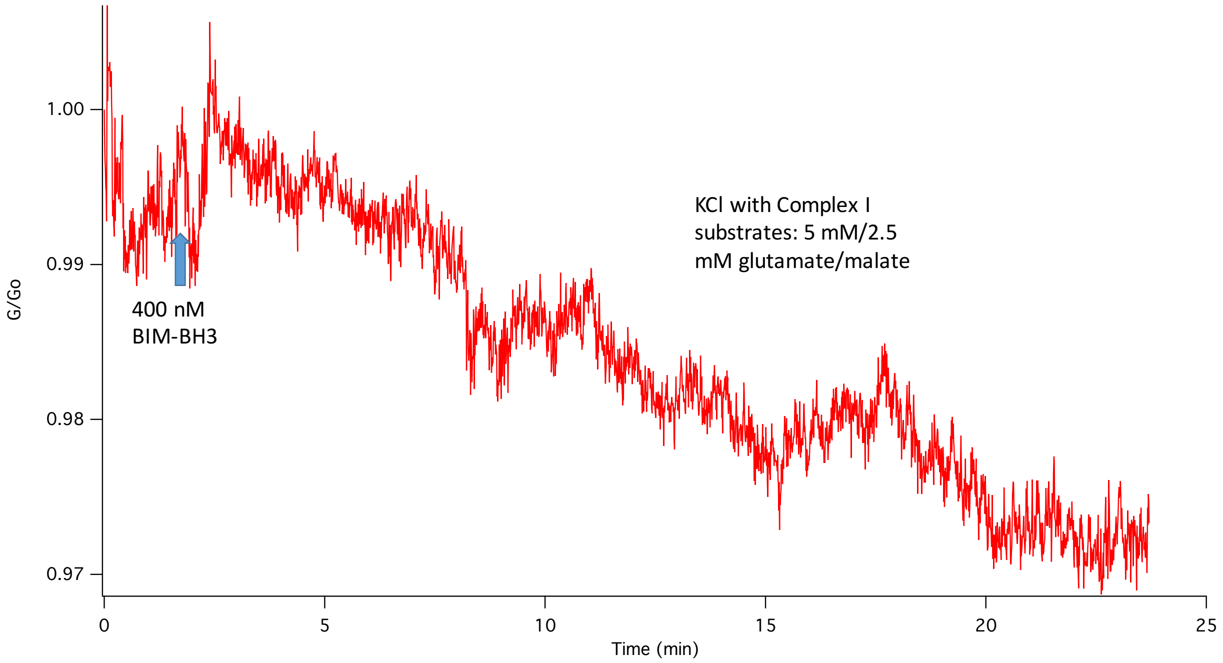

Supplementary Figure 12: We also performed an experiment with complex I substrates 5mM glutamate/2.5 mM malate in KCl buffer. 400 nM BIM addition causes a reduction in graphene conductance (or acidification). HeLa mitochondria were used.

References:

1. Gao, X. P. A., Zheng, G. & Lieber, C. M. Subthreshold Regime has the Optimal Sensitivity for Nanowire FET Biosensors. *Nano Lett.* **10,** 547–552 (2010).
2. Ferrer-Anglada, N., Kaempgen, M. & Roth, S. Transparent and flexible carbon nanotube/polypyrrole and carbon nanotube/polyaniline pH sensors. *Phys. Status Solidi Basic Res.* **243,** 3519–3523 (2006).
3. Mailly-Giacchetti, B. *et al.* pH sensing properties of graphene solution-gated field-effect transistors. *J. Appl. Phys.* **114,** 084505 (2013).
4. Rajan, N. K., Routenberg, D. a & Reed, M. a. Optimal signal-to-noise ratio for silicon nanowire biochemical sensors. *Appl. Phys. Lett.* **98,** 264107–2641073 (2011).
5. Gao, X. P. A., Zheng, G. & Lieber, C. M. Subthreshold Regime has the Optimal Sensitivity for Nanowire FET Biosensors. *Nano Lett.* **10,** 547–552 (2010).
6. Haines, T. H. & Dencher, N. a. Cardiolipin: A proton trap for oxidative phosphorylation. *FEBS Lett.* **528,** 35–39 (2002).
7. Kagan, V. E. *et al.* Cytochrome c acts as a cardiolipin oxygenase required for release of proapoptotic factors. *Nat. Chem. Biol.* **1,** 223–232 (2005).
8. Ryan, J., Brunelle, J. K. & Letai, A. Heightened mitochondrial priming is the basis for apoptotic hypersensitivity of CD4+ CD8+ thymocytes. *Proc. Natl. Acad. Sci.* **107,** 12895–12900 (2010).
9. Letai, A. *et al.* Distinct BH3 domains either sensitize or activate mitochondrial apoptosis, serving as prototype cancer therapeutics. *Cancer Cell* **2,** 183–192 (2002).
